# A holistic and sustainable approach to public health staffing and workforce development

**DOI:** 10.3389/fpubh.2025.1493858

**Published:** 2025-01-21

**Authors:** Paulani Mui, Ruth Maiorana, Beth Resnick

**Affiliations:** ^1^Department of Health Policy and Management, Johns Hopkins Bloomberg School of Public Health, Baltimore, MD, United States; ^2^Maryland Association of County Health Officers, Baltimore, MD, United States

**Keywords:** public health workforce, staffing, retention, recruitment, worker wellbeing

## Abstract

Public health in the United States faces a continuous cycle of “neglect, panic, repeat.” As seen with 9/11, H1N1, and COVID-19, public health emergencies create a flurry of attention and resources, but once the crisis passes, focus quickly shifts to other matters until the next emergency, when the cycle repeats. This leaves the nation's public health system chronically under-resourced and ill-equipped to respond, resulting in a strained workforce that must remain nimble. Maintaining responsiveness to community needs requires a sustainable system with adequate worker supports. This perspective discusses findings from an assessment of short-term COVID-19 investments on long-term U.S. public health workforce needs, highlighting the need for a holistic approach to staffing and workforce development. Temporary staffing addressed immediate response needs, but presented challenges such as difficulties transitioning temporary staff into permanent roles and cohesively integrating temporary staff into ongoing agency operations, which often inadvertently increased administrative burdens on existing staff, exacerbating burnout and dampening morale. Ensuring a sustainable workforce necessitates innovative recruitment and retention strategies. Recommended strategies include holistic recruitment efforts in collaboration with community and academic partners, enhanced leadership training, staff compensation reviews, flexible work arrangements, and worker wellbeing initiatives. These findings guided the creation of the Putting Our People First Discussion Guide to empower agencies to engage workers in collective dialogue to improve workforce mental health, wellbeing, and retention. Bolstering a culture of worker wellbeing and retention alongside sustained funding and infrastructure is critical for the nation's public health agencies to effectively address current and future challenges.

## Introduction

Understaffing is a chronic public health challenge; loss of public health positions since the Great Recession has been well documented (1). The 2020 Staffing Up project estimated a shortage of 80,000 staff across the nation's state and local public health departments (2), and the 2021 Public Health Workforce Interest and Needs (PH WINS) survey indicated an acceleration of staffing challenges (3). COVID-19 relief funds allowed many public health agencies to hire temporary staff to meet immediate pandemic response needs. However, temporary staffing often led to unintended consequences on the existing workforce. Additionally, many temporary positions were lost after pandemic funds ran out, highlighting the need for ongoing investment and sustained attention to sufficient staffing.

Federal investment in public health workforce and infrastructure via the American Rescue Plan Act of 2021 and the Centers for Disease Control and Prevention's Public Health Infrastructure Grants (PHIG) program offers a window of opportunity to bolster the public health workforce by addressing longstanding recruitment and retention challenges in state and local public health agencies (4). Such efforts should include institutionalizing policies and practices to promote and prioritize employee recruitment, retention, safety, growth, and mental health and wellbeing.

The U.S. Surgeon General's 2022 Framework for Mental Health and Wellbeing in the Workplace helped build national awareness regarding the need to prioritize workplace mental health and wellbeing (5). The framework called for workplaces to ensure the following five essentials for a healthy workforce: protection from harm; connection and community; work-life harmony; mattering at work; and opportunities for growth (5). Achieving a healthy workforce requires agencies to not only foster a supportive work culture where employees feel valued and heard but also implement inclusive workplace practices that promote employee physical and mental health, advance career development, and sustain work-life balance.

Aligning with the Surgeon General's framework, a holistic approach to public health workforce development—prioritizing continuous learning, technical and leadership skills development, and supports for employee mental health and wellbeing—can help facilitate greater employee engagement and mitigate burnout (6). This in turn can help drive innovation, productivity, and adaptability and improve employee job satisfaction and retention rates.

In this perspective, we will discuss three thematic areas that evolved from our research and key informant interviews with the public health workforce: (1) the benefits and challenges of temporary staffing via COVID-19 relief funding; (2) additional pandemic-related staffing challenges; and (3) strategies health departments have employed to extend staff capacity and transition temporary staff toward a permanent workforce. We then share our response to these findings through our development of an annotated discussion guide for agencies to engage workers in improving worker wellbeing and retention; and the case for why a holistic approach to workforce development is needed to sustain a strong public health workforce.

## Methods

Between May 2023 to September 2023, our research team conducted 10 stakeholder group discussions with over 80 individuals from 10 different public health associations and state and local public health agencies. To further triangulate findings, we conducted four additional key informant interviews with six public health professionals from four health departments. The discussions and interviews focused on the current state of the workforce and staffing strategies to build capacity and ensure sustainability.

Participating organizations included the Council of State and Territorial Epidemiologists (CSTE), Association of State and Territorial Health Officials (ASTHO), National Association of County and City Health Officials (NACCHO), Big Cities Health Coalition (BCHC), National Association of Local Boards of Health (NALBOH), State Associations of County and City Health Officials (SACCHOs) Council, Maryland Association of County Health Officers (MACHO), Washington State Association of Local Public Health Officials (WSALPHO), Wisconsin Association of Local Health Departments and Boards (WALHDAB), and the Public Health Accreditation Board's (PHAB) 21st Century Learning Community (21C) members. Thematic analysis of the group discussions and key informant interviews yielded common themes around the benefits and challenges of using COVID-19 relief funds for temporary staffing.

### Benefits and challenges of temporary pandemic staffing

Temporary COVID-19 investments provided the ability for public health agencies to more quickly onboard contract or temporary employees and introduced many young professionals to governmental public health. Yet, use of contract or temporary staff often led to unintended consequences, as these short-term hires often had higher salaries, more flexible remote work options, and less administrative authority and responsibility than existing staff. These differences often led to increased administrative burdens on full-time staffers as well as pay and remote work inequities that exacerbated demoralization, devaluation, and turnover among existing staff. Further, challenges in transitioning temporary or contract roles into full-time positions emerged due to a number of issues such as limits on the number of full-time agency staff positions allowed, stringent long-term funding requirements to establish new full-time positions, and in some states, specific restrictions from either the governor or state legislature prohibiting use of COVID-19 funds for staffing.

Additional COVID-19 temporary funding challenges included a lack of capacity to track COVID-19 relief funding usage; inability or delay in receiving the temporary COVID-19 funds (particularly at the local level); sustainability concerns in hiring permanent staff or making other long-term changes given the short-term nature of the COVID-19 funding; and restrictions on use of COVID-19 funds specific to department-wide infrastructure (e.g., inability to use COVID-19 funding for department-wide technology support). A general misalignment of federal funding aims and operational constraints was also discussed. For example, a subset of the federal COVID-19 workforce and infrastructure investments aimed to increase full-time public health staff; however, as noted above, some jurisdictions specifically disallowed the use of COVID-19 funding to hire full-time employees, thus leaving contract temporary staff positions as the only option.

### Other staffing challenges accelerated by the COVID-19 pandemic

Staff turnover in many public health agencies was accelerated by the rise of remote work opportunities that emerged from pandemic-related upstaffing. These new remote positions often offered geographic flexibility, professional advancement, and higher pay, enticing many public health agency employees to leave their roles. For example, many of the new remote staff members at national public health organizations (e.g., NACCHO, ASTHO, CSTE, etc.) were former public health agency employees. Within states, we heard of current public health employees leaving lower-paying local health departments for higher-paying and often more advanced remote roles at other local agencies or the state health department. Anecdotally, this trend was particularly challenging for remote and rural local health departments, which already typically face more difficulties in filling vacancies.

Compounding the turnover issue, key informants shared a range of challenges related to governmental hiring processes. Such challenges are not new; however, barriers to hiring became more problematic with an increased exodus of employees during the pandemic. The hiring challenges that were shared varied depending on the governance and structure of public health agencies, particularly the level of authority health departments had over their own hiring processes. For example, some agencies reported struggling with lengthy and complex hiring processes controlled by state, city, or county level human resource (HR) departments, leaving the health department with little to no autonomy over their hiring processes. In some jurisdictions, standardized HR job descriptions, which cannot be customized, had to be used for vacancies. For example, a generic job description for a Program Manager Level 1 position might be posted without specifying the program area or desired skills and experience, making targeted recruitment and the application review process more challenging. Lastly, jurisdiction-specific challenges, such as collective bargaining agreement requirements and civil service exams, were noted as recruitment barriers, particularly for applicants without prior government experience.

### Strategies to build workforce capacity

Key informants were optimistic about leveraging current federal public health infrastructure funding opportunities (e.g., PHIG) and partnerships with schools and programs of public health to enhance full-time employee recruitment efforts. Strategies currently being implemented in some agencies included internship and fellowship programs, along with joint recruitment initiatives in partnership with academic institutions. In some cases, these programs were designed with the intent to provide a seamless pipeline for participants to transition into full-time positions. Additionally, PHIG-funded Workforce Development Directors gave health departments the opportunity to have a dedicated leader focused on developing and implementing sustainable workforce solutions, in addition to addressing critical challenges in recruitment, retention, and employee wellbeing.

Building on these efforts, other commonly mentioned strategies focused on enhancing agencies' recruitment and hiring processes. Examples cited included collaborating with HR departments to gain earlier access to applicants and streamline communication with applicants, as well as strategically leveraging social media platforms such as LinkedIn, Indeed, and others to more effectively target candidates. By crafting compelling job descriptions and incorporating testimonials from current employees, agencies aimed to better highlight the benefits and impact of public health careers and attract stronger applicants (7).

Beyond recruitment, retention emerged as another key focus area. Strategies included revamping hiring and onboarding processes to help new employees feel more connected to the organization's mission and build community with department leadership and their colleagues. Other efforts that have been initiated included employee mentorship and recognition programs, staff advancement opportunities, and leadership training on critical topics such as mental health awareness, crisis management, and trauma-informed response.

Advancing workforce capacity also requires prioritizing worker wellbeing supports and resources, particularly in light of current malaise in the workforce stemming from pandemic-induced trauma and stress. Specific strategies shared included peer support groups, team-building and bonding activities, and recognition for service. Such efforts were emphasized as important ways to operationalize worker support as a fundamental component of public health infrastructure.

### Developing a discussion guide to advance workforce wellbeing

Building on the suggested strategies shared and leveraging funding from the CDC Foundation, we partnered with PHAB to develop an annotated discussion guide to engage public health agency leadership and staff on ways to collectively identify and address worker needs and enhance worker health, wellbeing, and workplace culture within the context of their own agency.

The discussion guide is designed to address each agency's unique needs, taking into account their specific structure, governance, and staffing challenges. Our research revealed a diverse range of issues, particularly related to staff turnover, telework policies, and disconnects between long-time employees and those hired after the COVID-19 pandemic. These findings underscored the need for targeted support to strengthen the workforce and the importance of allowing agencies the flexibility to adapt the discussion guide to their unique circumstances. Rather than presenting a one-size-fits-all approach, the guide offers a pathway for agency leadership to engage staff in meaningful dialogue to collectively identify their agency's core priorities and needs, as well as any current challenges their staff are facing. The guide includes critical questions to assess key elements, such as leadership support and organizational readiness, to gauge whether an agency is ready to move forward with efforts to advance worker wellbeing. In addition, the guide offers a pre-assessment tool to assess the agency's baseline and help guide next steps.

The guide also provides a suggested implementation roadmap that agencies can tailor to their specific needs. For example, an agency that lacks a dedicated workforce development committee might be seeking to develop a more intentional approach to workforce wellness through smaller, short-term improvements and therefore might want to follow the roadmap in full. Whereas an agency that already has workforce development initiatives underway with sufficient funding sources might be looking to find ways to sustain and evaluate progress going forward and therefore might skip ahead toward the end of the roadmap. However an agency decides to utilize the roadmap, its implementation will ultimately vary depending on agency-specific contextual factors such as agency size, staffing, nature of work, and available resources.

The discussion guide cannot be a one-time, single-use tool, but rather needs to be part of a continuous process to support worker wellbeing and retention for the long term. This need has also been recognized by the field, and thus we are engaging with ongoing efforts to advance and support the public health workforce, including ASTHO's Public Health—Hope, Equity, Resilience, and Opportunity (PH-HERO) initiative (8) designed to improve workforce wellbeing in state and territorial public health agencies and activities under PHIG grant funding. The discussion guide, as well as a selected assortment of workforce health and wellbeing resources targeted to public health agencies, can be accessed on the Putting Our People First: Prioritizing Public Health Worker Wellbeing webpage (9). This page was developed in partnership with the Johns Hopkins P.O.E. Total Worker Health^®^ Center, one of 10 academic Centers of Excellence for Total Worker Health funded by the National Institute for Occupational Safety and Health (10).

This guide is an important first step toward advancing workforce wellbeing as a fundamental component of public health infrastructure. Future efforts include piloting the guide in several state and/or local agencies and incorporating feedback from leadership and staff to refine the guide in alignment with agency needs and inform the development of supplementary tools such as checklists, case study narratives, and best practices to bolster impact and provide support and structure for discussion guide implementation. The guide aims to help agencies foster the development of workforce retention strategies to improve organizational structures and processes to advance a culture of worker wellbeing and retention while also helping state and local public health agencies achieve related workforce goals.

## Discussion

Efforts to strengthen the public health workforce have traditionally focused on recruitment, diversity, training, and skill development. As an example, PHAB public health agency accreditation workforce development plans primarily focus on planning and delivery of departmental trainings and often lack specific strategies for how to address workforce gaps (11). While all aforementioned elements are undeniably important, a broader context that also emphasizes worker wellbeing as a core component of the public health infrastructure is needed. A happy, healthy, and committed workforce can yield better organizational performance, productivity, and retention (5). Further, the key to a strong workforce lies in the ability not only to attract talent, but also to retain and nurture strong and talented workers. This necessitates a holistic approach that emphasizes workplace wellbeing as a fundamental aspect of workforce development and mission sustainability. By adopting such a perspective, we acknowledge and recognize the interconnectedness of employee wellbeing and organizational success in advancing the public's health. When coupled with sufficient funding and resources, we can ensure a well-prepared and equipped state and local public health system that can readily respond to public health emergencies.

Strategic partnerships and collaborations among leadership and staff play a pivotal role in changing the approach to workforce development. It is worth noting that this holistic approach to workforce development is increasingly gaining attention, not only in the public sector but also in the private sector, where the appointment of chief wellness officers underscores a growing recognition of the importance of employee wellbeing in achieving organizational objectives (12). Ensuring a sustainable and effective public health workforce necessitates prioritizing the people within. As U.S. Surgeon General Vivek Murthy noted, “a healthy workforce is the foundation for thriving organizations and healthier communities” (13).

## Data Availability

The data presented in this article may be available upon request subject to funding organization approval and compliance with participant confidentiality requirements.
